# Potentiality of *Moringa oleifera* as a Nutritive Ingredient in Different Food Matrices

**DOI:** 10.1007/s11130-022-01023-9

**Published:** 2022-11-11

**Authors:** Carla Trigo, María Luisa Castelló, María Dolores Ortolá

**Affiliations:** Institute of Food Engineering for Development, Universitat Politècnica de València, Camino de Vera S/N. 46022, Valencia, Spain

**Keywords:** *Moringa oleifera*, Sensory characteristics, Food ingredient, Technological properties, Food matrices

## Abstract

Given the growing interest of today's society in improving the nutritional profile of the food it consumes, industrial food reformulation is booming. In this sense, due to its high yield, good adaptation to climate change and high nutritional potential, *Moringa oleifera* may be an alternative means of fortifying products, in order to improve different food matrices. The different parts of this plant (leaves, seeds, flowers, pods, roots…) can be marketed for their nutritional and medicinal attributes. In this analysis, various scientific studies have been compiled that evaluate the potential of *Moringa oleifera* in terms of its incorporation into food matrices and its influence on the final sensory characteristics. In general, the incorporation of different parts of moringa into products, such as bread, pastries, snacks and beverages, increases the nutritional profile of the product (proteins, essential amino acids, minerals and fiber), the dried leaf powder representing an alternative to milk and eggs and helping vegans/vegetarians to consume the same protein content. In the case of dairy and meat products, the goal is to improve the antioxidant and antimicrobial capacity. In every food product, adding high concentrations of moringa leads to greenish colorations, herbal flavors and changes in the mechanical properties (texture, hardness, chewiness, volume and sponginess), negatively impacting the acceptance of the final product. This bibliographic review highlights the need to continue researching the technological properties with the dual aim of incorporating different parts of moringa into food matrices and increasing consumer familiarity with this product.

## Introduction

Despite growing at a slower pace, world population is expected to reach 9.7 billion in 2050 and could peak at nearly 11 billion around 2100 [1]. To accommodate this number, current food production will need to nearly double. This is a significant challenge for society, which it must accomplish while trying to follow most of the sustainable development goals [2]. The partial replacement of meat protein by vegetable protein may be a possible means of reducing the carbon footprint. Moreover, consumers are becoming increasingly aware of their fat intake and concerned about the products they include in their diets, because lifestyle habits and a diet rich in saturated fats, cholesterol, and salt have been shown to be risk factors for cardiovascular disease [3]. Bearing all these factors in mind, it is necessary to look for more sustainable and healthier products, which contribute to enhancing welfare and achieving ecological balance.

*Moringa oleifera* is a rapidly developing tropical crop, little known in developed countries but widely cultivated in Africa, Central and South America, Sri Lanka, India, Mexico, Malaysia, Indonesia and the Philippines since ancient times [4]. This crop has great industrial potential and is also well adapted to climate change because it needs little water and has few agricultural requirements, as was recently reported by the authors [5]. In addition, each one of its parts has significant nutritional properties, in particular their content in proteins, fiber, antioxidants [6, 7] and minerals. Thus, the leaves are used as nutritional supplements, the seeds for water purification, the oil as a biofuel, the trunk as a gum producer, the flowers as a source of honey, and every part of the plant can also be used for therapeutic purposes [8]. Remarkably, the oil produced from the seeds has a similar level of C18:1 to olive oil [9]. Moreover, in the developing countries, sources of vitamin A, such as drumstick leaves, are invaluable in the struggle to overcome the problem of vitamin A deficiency [10]. Hence, *M. oleifera* trees are being promoted as a dual solution to mitigate the impacts of climate change, while also providing an alternative source of income for families since these trees are easy to plant and do not require much maintenance [5].

The food industry might offer traditional products fortified with sustainable sources, such as the different parts of moringa, promoting the consumption of this plant. However, there is a lack of knowledge as regards the potential uses of moringa in the area of food fortification [11]. To that end, the objective of this paper is to collect recent information obtained from scientific references where moringa has been incorporated into a variety of food matrices (bread, bakery products, snacks, beverages, dairy and meat products) in order to discover its effect on technological properties and sensory acceptance.

## Application in Different Food Matrices

### Bread, Bakery Products and Snacks

Despite its low nutrient content, bread is a staple food the world over due to its high-energy input. Micronutrient deficiency is increasing among children and pregnant women in Africa and other developing countries [12]. In order to know how different parts of moringa contribute to the nutritional enrichment of bread, an extensive search for the information published in scientific articles over the last 10 years has been conducted and the results collected in Table 1. Specifically, seed flour and dry moringa leaf powder are mainly used to fortify bread dough prepared from either wheat flour or in combination with other flours. Moreover, this replacement can be a good alternative means of elaborating gluten-free bread intended for coeliacs, it being a good way to provide appropriate levels of minerals, proteins, phenols and other nutrients. The concentrations used to fortify bread with dry leaf powder and seed flour range from 1–15 to 1–5%, respectively. It is worth noting the viability and potentiality of the use of seed flour because it is just as rich in protein as the leaf [13]. Most of the authors highlight the improvement in the protein and fiber content of moringa*-*rich bread; in some cases, a decrease in moisture is also reported together with the subsequent shelf-life extension [14, 15]. However, negative characteristics are also registered, mainly related with mechanical properties (lower height, less volume and sponginess than the control) [14, 16–18]: this is probably due to gluten deficiency as a result of adding a greater proportion of moringa in the bread dough, as stated by some authors [16, 17]. Other authors conclude that in addition to gluten deficiency, the antimicrobial properties of moringa leaves have a significant impact on yeasts; this affects the structure of bread, as it is less rich in alveoli [14]. Also, concentrations of over 5% of seed flour and approximately 10% of dry leaf powder lead to low sensorial acceptance due to the dark green color, the herbal taste and the increased hardness of the bread.

**Table 1:** Moringa fortified breads

Part of the moringa	Moringa content	Beneficial features	Problematic features	Ref
Dried leaf powder	Wheat flour: Moringa powder ratio: 99:1, 98:2, 97:3, 96:4 and 95:5	Increases the content of protein and crude fiber and reduces the moisture content	Height and volume decrease in bread. Increasing the concentration of moringa reduces consumer acceptance	[16]
	Wheat flour: Moringa powder ratio:99:1, 98:2, 97:3, 96:4 and 95:5	Reduces humidity, prolonging the shelf life of the product, increases the content of proteins, carbohydrates and crude fiber	Not such a fluffy texture, lower height and volume due to the antimicrobial properties of moringa leaf	[14]
	Wheat flour: Moringa powder ratio:97.5:2.5, 95:5, 92.5:7.5 and 90:10	Increase in fat, minerals, crude fiber and protein. Decrease in humidity by prolonging the shelf life of the product	Concentrations of 10% affect consumer acceptance as a result of the dark green color of the bread and the herbal flavors	[15]
	Wheat flour: Moringa powder ratio:97.5:2.5, 95:5, 92.5:7.5 and 90:10	Nutritional improvement in phenol content and antioxidant activity. 2.5% and 5% have little influence on the taste of the bread	Decrease in the volume of bread caused by increasing the concentration of moringa. Concentrations of 10% affect consumer acceptance	[17]
	Wheat flour: Moringa powder ratio: 95:5, 90:10	Increase in protein content, iron and fiber. The conclusion is that brown bread would be more suitable for fortification	Higher concentrations limit acceptance due to increased hardness and the use of a lightening agent to mask the dark color is proposed	[19]
	Wheat flour: Moringa powder ratio: 95:5, 90:10 and 85:15	Increase in proteins, minerals and crude fiber content	Higher concentrations provide more grainy breads	[20]
Seed flour	1–15% of wheat flour replacement	Increase in protein content, fiber, and appearance comparable to control	Not such a soft, fluffy texture as the control sample	[21]
	Wheat flour: Moringa seed powder 95:5, 90:10, 85:15 and 80:20	Increase in protein content, fiber and fat content increases the palatability of the product and lowers humidity so there is a longer shelf life	Texture not as soft and fluffy as the control sample	[18]

Moringa may also be incorporated into other bakery products which, in spite of being less significant as regards people’s diets than bread, are widely consumed by all age ranges in different societies. In the last few years, moreover, industry has mass produced them with processed egg products, using “*trans*” fats and a high sugar content; the objective was to offer the products at low prices, which has led to an increase in the consumption of these kinds of industrial pastries that, in turn, has resulted in chronic diseases, such as obesity and cardiovascular diseases [22]. Industry is conscious of the need to improve the nutritional profile of these products and is making a great effort to develop healthier bakery foods, using natural products useful for disease prevention. In this regard, Table 2 has compiled data obtained from scientific articles over the last eight years dealing with the fortification and, therefore, nutritional improvement of biscuits, cakes, brownies and muffins that use different parts of moringa. As can be seen, dry leaf powder (1–20%), seed powder (10–30%) and, to a lesser extent, leaf extract (15–30 mL) replaced part of the wheat flour in cookies, muffins, brownies, etc. The beneficial properties of the fortification of these kinds of products are related with an increase in the nutritional content, above all in protein and fiber, and also with a greater antioxidant activity. In the case of vegetarians, for example, moringa leaves may be an alternative to milk and eggs in cookies that would enable them to consume the same content of proteins [23, 24]. It is noticeable that high concentrations of seed powder, around 20%, resulted in a good sensory acceptance as a result of the characteristic flavor imparted by the seed, which is very similar to the taste of the nut [21, 25]. However, the reformulation of bakery products also has some drawbacks, especially when 7–20% moringa is incorporated into these matrices; as a consequence, they exhibit greater compaction, unwanted odors and herbal flavors and a greenish coloration, all of which implies consumer rejection. These technological shortcomings can be overcome by coating the product with a chocolate layer, as reflected by these authors [23, 24], adjusting the amount or selecting the appropriate baking powder, among other strategies.

**Table 2:** Moringa-fortified bakery products

Part of the moringa	Moringa content	Bakery product	Beneficial features	Problematic features	Ref
Dried leaf powder	Wheat flour: Moringa powder ratio: 99:1, 98:2 and 97:3	Cookies	Higher protein and fiber content compared to the control sample	Color perceived by consumers as a sign of deterioration and led to rejection. However, it was more readily accepted with a cover over it	[23]
	Wheat flour: Moringa powder ratio: 95:5	Cookies	Nutritional improvement and good consumer acceptance	Consumers prefer a chocolate topping that provides more sweetness and disguises the greenish color of the cookie	[24]
	1–7% of wheat flour replacement	Muffins	Increasing concentration led to greater free radical removal activity and lower product hardness	The height of the product decreased in line with an increase in concentration. Concentrations of 7% led to reduced acceptance in taste and texture	[22]
	1–10% of wheat flour replacement	Muffins	Nutritional improvement of the product compared to the control sample	Concentrations greater than 10% made the product compact and caused an undesirable greenish coloration	[26]
	2, 4, 6, 8 and 10 g respectively with wheat flour	Cake	Higher content of proteins, fiber and essential amino acids compared to the control sample	Higher concentrations would be limiting due to color	[27]
	Wheat flour: Moringa powder ratio: 95:5, 90:10	Brownie	Higher ash content and lower lipid content compared to the control sample	Higher concentrations would be limiting due to color and flavor	[28]
	1–12% of wheat flour replacement	Muffins	Higher content of proteins and other macro and micronutrients compared to the control sample	Need for more research into the optimal use of leaves and other parts of the plant so that there is a greater application of the plant on the product	[11, 29]
	1–20% of wheat flour replacement	Cookies	Nutritional improvement of the product compared to control sample	Concentrations of 10% were comparable with the control; with those of 20%, however, only color and aroma were penalized and higher concentrations were not accepted	[30]
Seed powder	10–30% of wheat flour replacement	Cookies	Concentrations of 20% were accepted by the consumer, highlighting the fact that the moringa seed that has a very similar taste to walnut	Not found	[21, 25]
Aqueous leaf extract	10–40% of wheat flour replacement (p/v)	Cookies	Increased nutritional content, texture, color and taste	If moringa leaf extract is greater than 20 mL, it reduces consumer acceptance	[31]

Other cereal-based products, such as the snacks (bars, squares and tortilla chips…), are appreciated by consumers for their taste. However, their nutritional profile is often very poor, since they have high amounts of sugars and fats, and are scarce in proteins and other nutritional compounds, such as antioxidants. In this regard, these food matrices have been submitted to different fortifications in terms of fiber, vitamins or proteins [32–36]. Table 3 collects the scientific studies published over the last few years in which moringa seed flour, and especially dry leaf powder, have been added to different snacks in widely differing quantities: 0.6–45% and 2.5–7.5%, respectively. As in bread and bakery products, there is a reported increase in the content of proteins, minerals, essential amino acids and fiber. In most cases, these snacks are aimed at specific sectors of the population, ones in which nutrient requirements are greater (athletes, elderly people, children with malnutrition, etc.) [32, 37]. Again, mechanical (hardness and swelling capacity) and sensorial properties (dark green color and astringent flavor) conditioned their acceptance [33, 35].

**Table 3:** Snacks fortified with moringa

Part of moringa	Moringa content	Type of snack	Beneficial features	Problematic features	Ref
Dried leaf powder	15–45% (dry blend basis)	Bar	Higher content of fiber, proteins, minerals and vitamins	Tough texture	[32]
	2.5–10% (w/w)	Bar with amaranth and wheat flour	Higher values of the four essential amino acids	Lower protein content than other formulations and higher content of fats and kilocalories	[38]
	0.6–1.2% (w/w)	Bar with mandioca	Combating malnutrition	Little familiarity with the product	[39]
	1–5% (w/w)	Tortilla chips with corn flour	Higher content of minerals and crude proteins and lower fat content	Little familiarity with the product, concentrations greater than 1% led to rejection	[33]
	1–2% (w/w)	Bar with corn flour and cassava root	Nutritional improvement (protein, moisture, ashes) compared to the control sample	Not found	[34]
	Pre-treated moringa leaf flour: black gram: corn flour in ratio 20:60:20	Square snack with black gram and corn flour	Higher protein and dietary fiber content and lower content of fat	Improvable texture, dark color and astringent taste limit consumer acceptance	[35]
Seed flour	Maize flour and moringa seed flour in ratios 97.5: 2.5; 95: 5; 92.5: 7.5 using 100 percent maize flour as reference	Square snack with corn flour	Higher ash, protein and fiber content	Low swelling capacity and bulk density	[36]
Sprout powder	0–26% respect to solid ingredients (65/35 solid ingredients/liquid ingredients) 0–19% respect to solid ingredients (75/25 solid ingredients/liquid ingredients)	Healthy snack bars	Optimal formulation: 18% solid ingredients (65/35 SI/LI): higher protein, ash and fat and lower total carbohydrate contents. Good source of B1 and B2 vitamins and minerals. Enhanced the contents of total amino acid, the relative content of unsaturated fatty acids (FA), *γ*-aminobutyric acid and total and individual glucosinolates. Good sensorial acceptance	Formulations containing 75/25 solid to liquid ratio exhibited worse consistency	[37]

### Beverages

These food matrices are easily consumed along with meals and so they are good vehicles for the delivery of nutritional supplements into the body [40]. Beverages are by far the most active category of functional foods due to: (i) their convenience and the possibility of meeting consumer demands as regards container contents, size, shape, and appearance; (ii) their ease of distribution and the fact that they are a better means of storage for refrigerated and shelf-stable products; and (iii) the fact that they represent a great opportunity to incorporate desirable nutrients and bioactive compounds since homogeneous blending is easily achieved [41, 42]. In this section, beverages made of raw materials of vegetable origin, such as tubercles, grains, seeds, fruits and herb leaves are considered. Protein is one of the most deficient components in this type of product and, therefore, the addition of different parts of moringa would improve their nutritional profile [43], giving rise to products rich in all-vegetable protein. Thus, Table 4 shows the studies carried out with this aim in mind, which might help to broaden the range of products for those who may either have some type of intolerances or follow a type of vegetarian diet. As can be seen, dry leaf powder, fresh leaf, leaf infusion and leaf aqueous extract were the most commonly used parts of moringa, in percentage ranges of 1.1–100%, 1.5–4.5%, 50%, 10–50%, respectively. An increase in the nutritional value, especially of the antioxidant capacity and the content of phenols and proteins, was the main beneficial aspect described. As for the problematic characteristics, the higher concentrations imparted a bitter taste due to the presence of catechin (phenolic compound that the moringa leaf possesses) and caused an undesirable greenish coloration, flavor degradations over time and a loss of quality if stored more than a week due to the ease with which moisture is absorbed. Some of the authors pointed out the need for more research into the incorporation of aqueous leaf extract into beverages [44] and the difficulty for some consumers to accept certain drinks due to the characteristic taste and color [43, 45].

**Table 4:** Drink fortified with moringa

Part of the moringa	Moringa content	Type of drinks	Beneficial features	Problematic features	Ref
Dried leaf	Moringa extract: 2 g moringa power leaf/300 mL water Blended: 50/50, 50/70, 50/90, 50/110 (v/v) moringa extract/Aloe vera juice	Moringa-*Aloe* *vera* blended drink	Acceptable sensory attributes. Formulations containing 70 mL and 90 mL *Aloe vera* juice proved as best product in terms of color, aroma, flavor, taste, mouth feel and overall acceptance	Increase in moringa percentage; titratable acidity of the blend increased	[46]
	0.5 g moringa powder: 50 mL of water	Infusion	High phenolic content	Difficulty on the part of consumers to accept the infusion because it is a new product with a characteristic flavor	[45]
	5, 10, 15, 20% in a blended juice of pineapple juice beetroot juice and moringa leaf juice	Blended juice of pineapple juice, beetroot juice and moringa leaf juice	Optimal concentration: 25:15:60 (beetroot:moringa leaves:pineapple) Can be stored for 10 weeks at refrigeration temperature and 6 weeks at room temperature with addition of sodium benzoate	Not found	[47]
	1.5–4.5% (w/w)	Pineapple, apple and banana smoothie	All-vegetable product rich in protein and good antioxidant capacity	Higher concentrations provided bitterness and undesirable greenish hues	[43]
	Infusion: 20 g moringa leaf power/150 mL ethanol 5–10-15 mL infusion/L wort	Malt drink of Melkassa-7 (yellow maize)	Decrease in dissolved oxygen level with an increase in extract concentration. Microbial growth inhibition was considerable at high concentrations of the leaf extract	A slight decrease with the extract concentration in the score of sensory attributes (color bitterness, sweetness, flavor and overall impression)	[42]
	40 g, 80 g and 120 g in 4 L of Zobo drink	Zobo functional drink	Increase in vitamins and minerals compared to the control sample	Need for more research in order to extend product life	[44]

### Dairy Products

Milk and its derivative products are generally not regarded as a rich source of particular bioactive ingredients, such as polyphenols and antioxidants, in spite of their many healthy and nutritious characteristics. Thus, health-conscious consumers are demanding more and more novel dairy products formulated using medicinal herbs or their extracts [48]. In this regard, Table 5 shows a compilation of recent publications in which dairy products have been reformulated using different parts of moringa. These studies focus on yogurt, curd and cheese. In the case of yogurt, [49–51] a concentration of 0.5–2% of dried moringa leaves was employed, whereas in the study carried out by Zhang et al. [52], the yogurt was supplemented with 0–0.2% moringa extract (ME; hot water extract, 100 °C, 30 min). Dhawi et al. [48] added 0.1 and 0.2% of *Moringa oleifera* seed flour to yogurt. Most of the beneficial features reported in these papers are linked to the maintenance of the sensory acceptance, the higher content of some amino acids (alanine acid, glutamine and tyrosine) and the enhanced antioxidant activity. Furthermore, moringa exerts control against pathogenic bacteria, such as *E. coli*, *S. aureus*, *L. monocytogenes* and *Salmonella *spp*,* without negatively affecting *L. rhamnosus* or *L. acidophilus* growth, also increasing the content in Ca, P, K, and Fe*.* However, a decrease in *L. rhamnosus,* lower viscosity and whiteness and less cysteine, methionine and histidine were found after five weeks of storage at 4 ºC. In most cases, a light green color was also registered. Moringa pod powder was incorporated into curd, consequently showing higher levels of vitamins A and C, iron, fiber and potassium than the control sample [53]. Labneh cheese [54], soft white cheese [51] and cream cheese [55] were prepared with 1–3% of dry moringa leaves or an ethanolic extract of dry leaves. As in previous cases, the nutritional value increased with acceptable organoleptic scores, although they had a lower degree of whiteness than the control.

**Table 5:** Dairy products fortified with moringa

Part of the moringa	Moringa content	Dairy product	Beneficial features	Problematic features	Ref
Dried leaf powder	2% milk was replaced for the MRS with 0.5–5% of moringa	yogurt	Sensory evaluation showed that not all yogurts were rated as being significantly different from the control, except in terms of appearance *M. oleifera* did not negatively affect the growth of *Lactobacillus rhamnosus* GR-1	A significant decrease in sensory evaluation was found after 5 weeks at 4 ºC	[49]
	17.09 g of dried moringa leaves per 1 L of yoghurt	banana, sweet potato or avocado yogurt	Sensory characteristics of moringa-banana yogurt were not significantly different from those of the control sample	Light green color. In general, lower scores in texture, flavor and overall quality	[50]
	0.5, 1, 1.5 and 2% (w/w)	Yogurt	0.5% was the best ratio and scored the highest for flavor as well as for taste Treatment imparted higher content of total solids, fats, total proteins, soluble nitrogen, total volatile fatty acids, acetaldehyde, diacetyl and lower pH than the fresh control sample. Yogurt showed the highest content of alanine acid, glutamine and tyrosine	Lower degree of viscosity and whiteness than the control sample. Less cysteine, methionine and histidine	[4]
	1, 2, or 3% (w/w)	Labneh cheese	Enrichment in minerals (Ca, Fe, Zn and Si) and vitamins (A, B1, B2 and E). Yeast & mold and coliform bacteria were not detected. Acceptable organoleptic scores during storage	Greener color, although this does not mean it is rejected since Labneh cheese is usually formulated with coriander& parsley and other herbs	[54]
	1, 2, or 3% (w/w)	Soft white cheese	1% moringa was the best ratio: good appearance, texture and flavor. Treatments imparted a higher content of glutamic acid, proline and leucine than the control. Nutritional value increased	Lower degree of whiteness than the control	[51]
Aqueous moringa leaf extract	0.05, 0.1, and 0.2%, w/v	Yogurt	Yogurt fermented using moringa extract maintains sensory acceptance and exerts positive health benefits because of increased *Lactobacillus acidophilus* proliferation and enhanced antioxidant properties	None	[52]
Seed flour	0.1% (M1) and 0.2% (M2) (w/w)	Buffalo yogurt	M2 yogurt had the highest values of total phenolic content and antioxidant activity and it exhibited significantly greater antibacterial activity against *E. coli, S. aureus, L. monocytogenes*, and *Salmonella* spp. M1 and M2 led to a higher content of Ca, P, K, and Fe	None	[48]
Pod powder	1.5% (w/w)	Curd	Higher levels of vitamins A and C, iron, fiber and potassium than the control sample	None	[53]
Ethanol extract of dried leaves	2, 3 and 4 g/100 g skimmed UF-retentate	Cream cheese	They can be used as a nutritional supplement and preservative agent	An increase in the leaf extract led to a greater degree of hardness, whereas cohesiveness followed the opposite tendency	[55]

### Meat Products and Their Plant-Based Analogues

Of the current consumer trends, one is the growing market for vegetarian and vegan foods; in recent years, this growth has been powered either by moral principles or because it is beneficial for both people’s health and the environment. In the human diet, protein intake is essential and, although there is a tendency to replace it with sources of plant origin, this comes mostly from animals [56]. In general, society consumes a large quantity of animal products, especially the young; in many cases, the production of these products (hamburgers, sausages, nuggets…) is based on low-cost meat with a high fat content. In addition, the impact of livestock on the environment is reflected directly and indirectly in the water, soil and air (generating greenhouse gases that increase the global temperature of the planet). Despite the high protein content of moringa, there are no scientific studies into the total substitution of protein in products traditionally made with protein of animal origin.

As regards the production of meat products, there is also a growing demand for natural effective antioxidants, because they are increasingly considered safer for consumption. Synthetic antioxidants, widely used in the food industry to lengthen the shelf life and improve the color and taste stability of meat products, have been considered in some studies to be a risk factor for degenerative diseases, such as cancer [57, 58]. As an alternative, the food industry is looking for natural sources of antioxidants, such as moringa. In this sense, Table 6 shows some studies that focus on analyzing the effect of the addition of moringa on various properties of meat products. As can be seen, the incorporation of moringa (aqueous extract of dried or fresh leaf, dried leaves, leaf powder, flower extract and seed flour) into meat products, such as sausages, chorizo, nuggets, minced meat and mortadella with raw material (beef, chicken, buffalo, pork and goat), is performed with the twofold purpose of inhibiting lipid oxidation [3, 59–62] and obtaining antioxidant and antimicrobial effects during storage, as recorded by these authors [63, 64]. Most of the authors agree that the great disadvantage is that high concentrations give rise to unwanted flavors and colorations and textural changes in the final product.

**Table 6:** Meat products and their plant-based analogues fortified with moringa

Part of the moringa	Moringa content	Meat product	Beneficial features	Problematic features	Ref
Aqueous dried leaf extract (20%)	Meat soaked in water (1:2 w/v) with 0.1–0.3 g extract/L	Atmosphere packaged raw beef	Inhibition of lipid oxidation	Higher concentrations gave unwanted flavors	[59]
	300–450-600 ppm equivalent *M. oleifera* leaves phenolics/100 g meat	ground pork patties	Inhibition of lipid without any adverse effects on sensory attributes	Higher concentrations gave unwanted flavors	[60]
Aqueous fresh leaf extract (15%)	1.0, 1.5 and 2% of aqueous solution in meat	Cooked ground buffalo meat	Significant antioxidant and antimicrobial effects improved the quality of the meat (tenderness, juiciness, prevention of discoloration)	Higher concentrations gave unwanted flavors	[63]
Dried leaves	0.25–1% (w/w)	Chicken sausages	Inhibition of microbial growth and lipid oxidation during cold storage (4 ºC)	0.75% and 1% additions produced a negative effect on sensory and textural attributes	[65]
Lyophilized flower aqueous extract	1—2%	Goat meat nuggets	Increase in the ash, dietary fiber and phenolic contents. Lower TBARS number	Low flavor score for treatment with 2%. Hunter green color and textural parameters were significantly affected	[62]
Leaf powder	1—3%	Veggie burger: (*Cucurbita moschata*), (*Erythrina edulis*) and (*Musa *spp.)	With 1%, microbial proliferation was reduced by 10%	Above 1%, organoleptic characteristics were negatively affected	[64]
Seed flour	1, 3 and 5%	Chicken mortadella	3 or 5% promoted a reduction in lipid oxidation during storage. 3% of moringa seed flour seemed to be ideal for chicken mortadella	The addition of 5% affected color parameters	[3]
Flower extracts	Aqueous (AE) or aqueous ethanol (AEH). (60:40, v/v) 1—2% of extracts	Cooked chicken nuggets	Improved the cooking yield and dietary fiber content without affecting the acceptance. Increased the lipid stability, odor score and shelf-life throughout 20 days of refrigerated storage	Extract at 2% increased the lightness and reduced redness, hardness, gumminess and chewiness	[61]

Some authors, in particular Madane et al. [61] and Verma et al. [62] recorded that the incorporation of flower extract increases the content of dietary fiber, ashes and phenolics and also improves the cooking yield of the final product. However, high concentrations of flower extracts (2%) led to an increase in the lightness and a reduction in the hardness, redness and chewability of nuggets.

## Conclusions

In this article, recently published scientific studies have been presented corroborating the potential of *Moringa oleifera* as an ingredient in different food matrices. It has been incorporated in most cases as dry leaf powder and seed flour to fortify breads, pastries (cookies, cakes, brownies, muffins) and cereal-based snacks. In those cases, all of the authors reported an improvement in the content of the proteins, essential amino acids, minerals and fiber of the final product. In some cases, an improvement in antioxidant capacity and a decrease in humidity have also been registered, thus extending the shelf life. In general, the negative consequences brought about by the antimicrobial properties of moringa that affect the action of yeasts are related to both the mechanical properties (lower height, less volume and fluffiness than control) and also to the sensory properties (dark green color and astringent flavor) that conditioned its acceptance.

Vegetable drinks are also food matrices fortified with moringa dried leaf powder, fresh leaf, leaf infusion or aqueous leaf extract, exhibiting an increase in nutritional value in every case, especially in terms of antioxidant capacity, and phenolic and protein content. However, higher concentrations imparted a bitter taste due to the presence of catechin, which produced an undesirable greenish coloration.

Dairy products, such as yogurt, curd and cheese, have also been fortified using different parts of moringa, giving rise to good sensory acceptance, a higher content in some amino acids (alanine acid, glutamine and tyrosine) and an improvement in antioxidant activity.

Finally, moringa was incorporated into meat matrices in very small proportions, the main objective of which was to search for its antioxidant and antimicrobial properties. In general, most authors agree that very small additions (1–2%) have an impact on changes in color, texture and flavor, as detected by tasters.

## Data Availability

Not applicable.
